# MIL-Derived Hollow Tubulous-Shaped In_2_O_3_/ZnIn_2_S_4_ Z-Scheme Heterojunction for Efficient Antibacterial Performance via In Situ Composite

**DOI:** 10.3390/nano14161366

**Published:** 2024-08-21

**Authors:** Jiao Duan, Hui Zhang, Jie Zhang, Mengmeng Sun, Jizhou Duan

**Affiliations:** 1 Key Laboratory of Advanced Marine Materials, Key Laboratory of Marine Environmental Corrosion and Bio-Fouling, Institute of Oceanology, Chinese Academy of Sciences, Qingdao 266071, China; duanjiao19981203@163.com (J.D.); zhanghui951227@163.com (H.Z.); duanjz@qdio.ac.cn (J.D.); 2 University of Chinese Academy of Sciences, 19 (Jia) Yuquan Road, Beijing 100049, China

**Keywords:** MIL-derived In_2_O_3_, hollow tubulous structure, Z-scheme heterojunction, photocatalytic sterilization, *Pseudomonas aeruginosa*

## Abstract

In this study, a hollow tubulous-shaped In_2_O_3_ derived from MIL (MIL-68 (In)) exhibited an enhanced specific surface area compared to MIL. To further sensitize In_2_O_3_, ZnIn_2_S_4_ was grown in situ on the derived In_2_O_3_. The 40In_2_O_3_/ZnIn_2_S_4_ composite (1 mmol ZnIn_2_S_4_ loaded on 40 mg In_2_O_3_) exhibited degradation rates of methyl orange (MO) under visible light (80 mW·cm^−2^, 150 min) that were 17.9 and 1.4 times higher than those of the pure In_2_O_3_ and ZnIn_2_S_4_, respectively. Moreover, the 40In_2_O_3_/ZnIn_2_S_4_ exhibited an obviously improved antibacterial performance against *Pseudomonas aeruginosa*, with an antibacterial rate of 99.8% after visible light irradiation of 80 mW cm^−2^ for 420 min. The 40In_2_O_3_/ZnIn_2_S_4_ composite showed the highest photocurrent density, indicating an enhanced separation of photogenerated charge carriers. Electron spin resonance results indicated that the 40In_2_O_3_/ZnIn_2_S_4_ composite generated both ·O_2_^−^ and ·OH radicals under visible light, whereas ·OH radicals were almost not detected in ZnIn_2_S_4_ alone, suggesting the presence of a Z-scheme heterojunction between In_2_O_3_ and ZnIn_2_S_4_, thereby enhancing the degradation and antibacterial capabilities of the composite. This offers fresh perspectives on designing effective photocatalytic materials for use in antibacterial and antifouling applications.

## 1. Introduction

Biofilm formed by marine microorganisms and their metabolites on the surfaces of marine facilities serve as carriers for the attachment of macroorganisms, leading to marine biofouling [1]. Marine biofouling is a significant factor affecting and constraining the development of marine industries. Currently, antifouling coatings containing biocides are the most commonly used method. However, the slow release of biocides may pose threats to the safety of marine ecological environments [2]. Therefore, the development of novel environmentally friendly antimicrobial and antifouling materials to replace toxic alternatives has been recognized as an inevitable choice for sustainable development [3]. Photocatalysis generates highly oxidative reactive radicals capable of effectively degrading organic compounds and disrupting microbial cell structures. Moreover, photocatalytic sterilization is environmentally friendly and does not pose issues related to biological resistance [4,5]. Since sunlight can penetrate up to 200 m in seawater, it can be fully received in the upper layers of the ocean where biofouling occurs. Therefore, photocatalytic technology holds significant potential for applications in marine antimicrobial and antifouling fields.

Indium oxide (In_2_O_3_) is a semiconductor material with a relatively narrow bandgap, attracting attention due to its good stability and conductivity [6,7]. However, conventional In_2_O_3_ photocatalysts often suffer from limitations such as a simple structure, small surface area, weak light response, and high recombination rate of photogenerated charge carriers, which restrict their further application in photocatalysis [8]. To enhance the photocatalytic performance of monolithic In_2_O_3_, researchers have explored various methods including elemental doping [9,10], morphology control [11], and the construction of heterojunctions [12,13], achieving significant improvements. Recently, In_2_O_3_ photocatalysts prepared via the pyrolysis of metal-organic frameworks (MOFs) have garnered significant attention. Liu et al. achieved the formation of rhombohedral corundum/cubic In_2_O_3_ by the direct annealing of NH_2_-MIL-68(In) in air atmosphere. Spectroscopic and photoelectrochemical tests demonstrated that this unique structure effectively accelerates the separation and transfer of photogenerated charges within In_2_O_3_ [14]. Yang et al. employed a simple oil bath method to grow CdZnS on shuttle-shaped mesoporous In_2_O_3_ derived from NH_2_-MIL-68. Under visible light irradiation, the photocatalytic hydrogen evolution rate was significantly enhanced. This improvement can be attributed to several factors: the derived In_2_O_3_ possessing mesopores which increase active sites, the introduction of ultrafine CdZnS nanoparticles reducing the bandgap of material, thereby enhancing the visible light response, and the construction of Type II heterojunctions which enhance the separation efficiency of photogenerated charge carriers in the composite material [15]. Research indicates that MOFs-derived In_2_O_3_ can retain the original framework structure of MOFs, while the resulting hollow structures and pores provide additional reactive sites for photocatalytic reactions, thereby enhancing photocatalytic activity. In situ composite semiconductor heterojunctions constructed with multi-site hollow framework MOFs derivatives can further improve photocatalytic activity [13,16].

Ternary metal sulfides, characterized by narrow bandgaps, good stability, and excellent electrical and optical properties, are considered promising photocatalytic materials with high performance and potential applications [17]. Among these, ternary metal sulfides with an AB_2_X_4_ structure exhibit excellent photocatalytic stability. Within the AB_2_X_4_ series, ZnIn_2_S_4_ stands out due to its narrower bandgap and absence of toxic metal ions, making it an excellent candidate for photocatalytic applications in the energy and environmental fields. ZnIn_2_S_4_ is commonly used to sensitize wide-bandgap semiconductor materials, enhancing the charge carrier separation efficiency of monolithic semiconductor materials through the construction of heterojunction-based multi-component photocatalytic systems. Moreover, the facile preparation method of ZnIn_2_S_4_ enhances its appeal as an excellent candidate for modified materials [18,19].

In this paper, the derived hollow tubulous-like In_2_O_3_ was synthesized through calcination. Compared to the MIL, the derived In_2_O_3_ exhibited a reduced bandgap, increased optical absorption threshold, and enhanced photoelectric response. The composite material based on hollow tubulous-like In_2_O_3_ with in situ-grown ZnIn_2_S_4_ exhibits significantly enhanced photosensitivity compared to individual In_2_O_3_ and ZnIn_2_S_4_. Among them, the 40In_2_O_3_/ZnIn_2_S_4_ (1 mmol ZnIn_2_S_4_ loaded on 40 mg In_2_O_3_) composite shows the highest performance. The application potential of In_2_O_3_/ZnIn_2_S_4_ composites for marine antibacterial and antifouling purposes was evaluated by measuring their capability to degrade MO dye and eradicate *Pseudomonas aeruginosa* (*P. aeruginosa*). The 40In_2_O_3_/ZnIn_2_S_4_ composite degraded 99.5% of 10 ppm MO under simulated visible light irradiation for 150 min, which is 17.9 times higher than In_2_O_3_ and 1.4 times higher than ZnIn_2_S_4_ alone. Additionally, the composite achieved a 99.8% antibacterial rate against *P. aeruginosa* after 420 min of light exposure, demonstrating a significant enhancement in photocatalytic performance compared to the individual photocatalytic materials. Electron Spin Resonance (ESR) studies indicate that both In_2_O_3_ and ZnIn_2_S_4_ can generate ·O_2_^−^ radicals, attributed to their conduction band potentials (*E*_CB_ = −0.59 V (In_2_O_3_), *E*_CB_ = −0.75 V (ZnIn_2_S_4_)) being more negative than the reduction potential of O_2_/·O_2_^−^ (−0.33 V vs. NHE). Additionally, only In_2_O_3_ can generate ·OH radicals due to its valence band potential (*E*_VB_ = 2.75 V) being more positive than the oxidation potential of H_2_O/·OH (*E*_VB_ = 2.40 V vs. NHE). However, the composite material 40In_2_O_3_/ZnIn_2_S_4_ exhibits increased production of both ·O_2_^−^ and ·OH radicals, suggesting the formation of a Z-Scheme heterojunction structure between In_2_O_3_ and ZnIn_2_S_4_, thereby enhancing the separation of photogenerated charge carriers. The composite material, prepared by the in situ growth of ZnIn_2_S_4_ on MIL-derived hollow tubular In_2_O_3_, demonstrates obviously enhanced degradation and antibacterial capabilities compared to pure In_2_O_3_ and ZnIn_2_S_4_, further expanding the possibilities for practical applications. Meanwhile, this advancement provides new insights for the development and design of novel, efficient photocatalytic antibacterial and antifouling materials.

## 2. Experimental Section

### 2.1. Synthesis of Rod-Shaped MIL-68 (MIL)

Using a solvothermal method, 5 mmol of indium nitrate (In(NO_3_)_3_) was dissolved in 35 mL of N,N-Dimethylformamide (DMF) to form solution A, while 6 mmol of phthalic acid (C_8_H_6_O_4_) was dissolved in 35 mL of DMF to form solution B. Solution A was then added dropwise to solution B under stirring at room temperature for 30 min. The resulting mixture was transferred to a high-pressure reaction vessel and subjected to a hydrothermal reaction at 100 °C for 12 h. Afterward, the product was centrifuged, washed several times with ethanol, and dried under vacuum at 80 °C for 12 h, yielding a white powder of MIL.

### 2.2. Synthesis of MIL-Derived In_2_O_3_ Photocatalyst

A certain amount of MIL powder was spread evenly in a crucible. The temperature was then ramped up at a rate of 10 °C/min until reaching 500 °C and maintained for 2 h, resulting in off-white In_2_O_3_ powder.

### 2.3. Synthesis of ZnIn_2_S_4_ Photocatalyst

In total, 1 mmol of ZnCl_2_, 2 mmol of InCl_3_, and 4 mmol of thiourea (TAA) were dissolved in 100 mL of deionized water. The mixture was stirred at room temperature for 1 h. Subsequently, the solution was heated and stirred in a water bath at 80 °C for 2 h. After cooling, the resulting mixture was centrifuged and washed several times with deionized water and ethanol. Finally, the material was dried under vacuum at 80 °C for 12 h to obtain the ZnIn_2_S_4_ photocatalyst.

### 2.4. Synthesis of In_2_O_3_/ZnIn_2_S_4_ Composites

**Figure 1:**
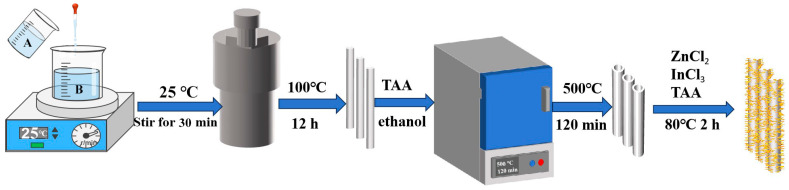
Synthesis process of the In_2_O_3_/ZnIn_2_S_4_ composites.

In total, 30, 40, 50, 60, and 300 mg of In_2_O_3_ were individually weighed and dispersed in 100 mL of deionized water with stirring for 30 min to ensure thorough dispersion. Subsequently, 1 mmol of ZnCl_2_, 2 mmol of InCl_3_, and 4 mmol of thiourea (TAA) were added sequentially to each dispersion, followed by stirring at room temperature for 1 h. The mixtures were then subjected to constant-temperature reactions at 80 °C in a water bath for 2 h. After cooling, the reaction products were collected by centrifugation, washed several times with deionized water and ethanol, and finally dried under vacuum at 80 °C for 12 h. The resulting materials were named as follows based on the amount of In_2_O_3_ used: 30In_2_O_3_/ZnIn_2_S_4_, 40In_2_O_3_/ZnIn_2_S_4_, 50In_2_O_3_/ZnIn_2_S_4_, 60In_2_O_3_/ZnIn_2_S_4_, and 300In_2_O_3_/ZnIn_2_S_4_. The synthesis steps of the materials are shown in the Figure 1 below.

### 2.5. Characterization

X-ray diffraction spectra was used to characterize the crystal structure of the prepared composites (XRD, Smart Lab, Rigaku Co., Tokyo, Japan); Scanning electron microscopy was used to observe the microstructure of the samples (SEM, JSM-7601F, NEC Co., Tokyo, Japan); and the species and distribution of elements were determined by energy dispersive X-ray spectroscopy (EDS, Oxford INCAx-sight, Oxford, UK). The microscopic bonding of the composites was further observed using transmission electron microscopy (TEM, JEOL 2100F, NEC Co., Tokyo, Japan); Ultraviolet-visible diffuse reflectance spectroscopy (UV-Vis DRS, U-3900H, Shimadzu, Kyoto, Japan) was used to observe the optical properties of the composites and calculate their bandgap information; The valence states of reactive elements were determined by X-ray photoelectron spectroscopy (XPS, ESCALAB 250Xi, Thermo, Waltham, MA, USA). The production of free radicals was detected by electron paramagnetic resonance spectroscopy (EPR, EMXplus, Bruker Co., Billerica, MA, USA; Ettlingen, Germany). 

### 2.6. Photoelectrochemical Test

The photoelectrochemical testing was conducted using a CHI660E electrochemical workstation (CHI660E, Shanghai Chenhua Instrument Co. Ltd., Shanghai, China). A three-electrode system was employed, with an Ag/AgCl electrode as the reference electrode, a Pt electrode as the counter electrode, and the prepared photoelectrode as the working electrode. The experiments were carried out in a 3.5 wt% NaCl solution to simulate seawater conditions. In addition, a xenon lamp (PLS-SXE300D, Beijing Perfect Light Co., Ltd., Beijing, China) was used as the simulated solar light illumination. The light was filtered through an AM 1.5 filter to obtain the simulated solar light and then adjusted to an intensity of 100 mW·cm^−2^. For the Mott–Schottky measurements, the open circuit potential of the system was first determined. Then, the Mott–Schottky plot was tested over a range of ±0.6 V with a frequency of 1000 Hz and an AC voltage amplitude of 10 mV.

### 2.7. Photocatalytic Degradation and Photocatalytic Antibacterial Performance

**Figure 2:**
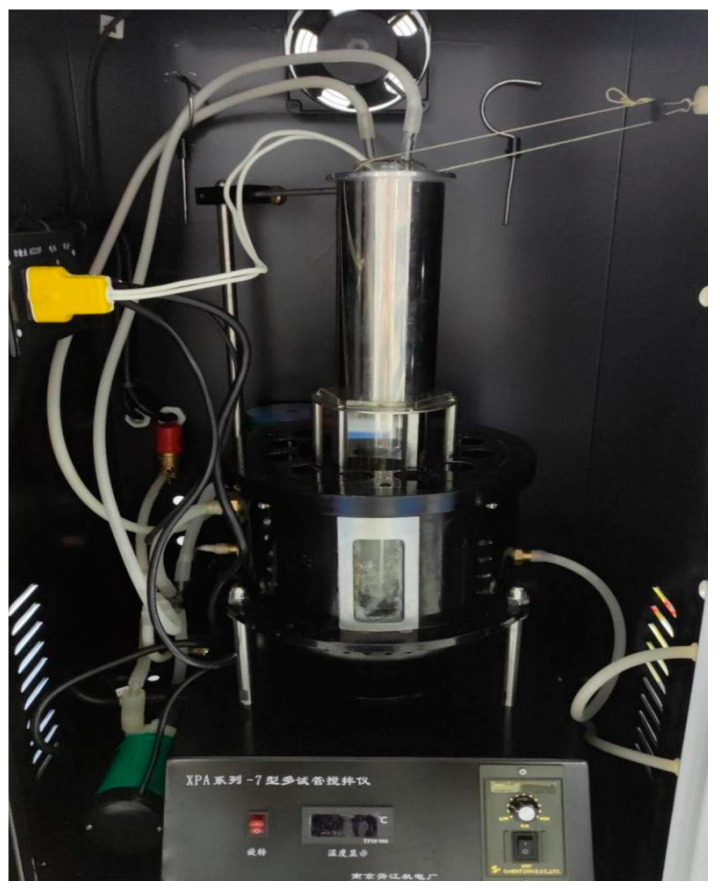
Internal structure of the photochemical reaction apparatus.

For the photocatalytic degradation of methyl orange (MO), 15 mg of the sample was weighed in a 50 mL quartz tube, followed by adding 50 mL of a 10 ppm MO solution; each sample was tested three times. The quartz tube was then placed into a photocatalytic reactor (Figure 2) and stirred for 60 min to achieve adsorption–desorption equilibrium. Subsequently, photocatalytic degradation reactions were conducted under 800 W xenon lamp irradiation (with a 420 nm cut-off wavelength filter and a light intensity of 80 mW·cm^−2^). Then, 1 mL solution of each sample was taken every 30 min during the reaction, and finally, the changes in MO absorbance were measured using an enzyme-labeler (YP-96C, Youyunpu, Weifang, China) to calculate the degradation rate.

The photocatalytic antibacterial experiments used *P. aeruginosa* (BNCC186070), commonly found in marine fouling biofilm. In total, 50 mg of the photocatalyst was added to a 50 mL quartz tube, followed by adding 49.5 mL of sterilized 0.1 M phosphate buffer saline (PBS), and each sample requires three parallel experiments. This was inoculated with 500 μL of an appropriately diluted bacterial suspension (5.9 × 10^8^ cfu·mL^−1^). The experiments were also conducted in a photocatalytic reactor with the same simulated light conditions. Prior to light exposure, the mixture was stirred for 60 min to achieve adsorption–desorption equilibrium in the dark condition. Then, 100 μL of the bacteria solution from the quartz tube was collected every 60 min. The antibacterial efficiency was determined by counting viable bacteria using the colony-counting method on a solid LB medium plate. The equipment used for photocatalysis is as follows:

## 3. Results and Discussion

### 3.1. Analysis of Physical Properties

**Figure 3:**
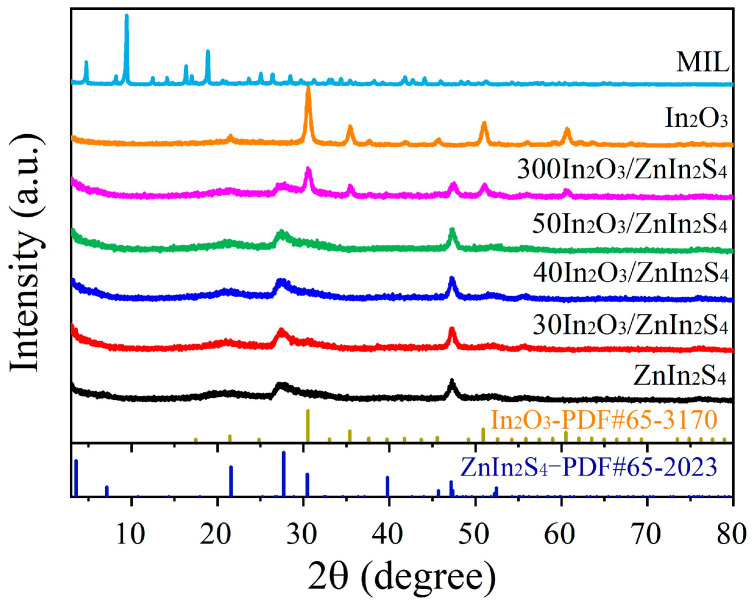
XRD patterns of MIL, In_2_O_3_, ZnIn_2_S_4_, and In_2_O_3_/ZnIn_2_S_4_ composites.

The XRD patterns of MIL, derived In_2_O_3_, ZnIn_2_S_4_, and different In_2_O_3_/ZnIn_2_S_4_ composites were determined, and the results are shown in Figure 3. It is evident that the peaks of the rod-like MIL template are sharp, indicating good crystallinity, and the peak patterns correspond well with those reported in the literature [20]. The diffraction peaks of the derived In_2_O_3_ at 2*θ* = 21.5°, 30.5°, 35.4°, 45.6°, 50.9°, and 60.6° correspond to the (211), (222), (400), (400), (440), and (622) crystal planes of cubic-phase In_2_O_3_ (PDF#65-3170), indicating that the material derived from MIL after high-temperature calcination is In_2_O_3_. The characteristic peaks at 21.6°, 27.7°, and 47.2° in the synthesized ZnIn_2_S_4_ correspond to the (006), (102), and (110) diffraction peaks of hexagonal-phase ZnIn_2_S_4_ (PDF#65-2023), confirming the successful synthesis of ZnIn_2_S_4_. The XRD analysis of the 300In_2_O_3_/ZnIn_2_S_4_ composite shows characteristic diffraction peaks of both In_2_O_3_ and ZnIn_2_S_4_, indicating the simultaneous presence of both materials. However, in the XRD spectra of 30In_2_O_3_/ZnIn_2_S_4_, 40In_2_O_3_/ZnIn_2_S_4_, and 50In_2_O_3_/ZnIn_2_S_4_, no distinct In_2_O_3_ diffraction peaks are observed. This suggests that In_2_O_3_ is heavily loaded with ZnIn_2_S_4_, resulting in the characteristic peaks of In_2_O_3_ being less pronounced.

**Figure 4:**
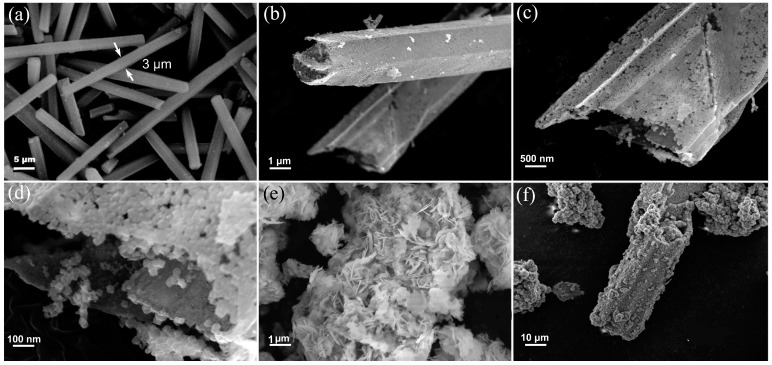

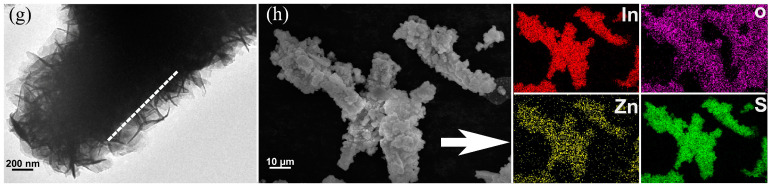
SEM images of MIL (**a**), In_2_O_3_ (**b**–**d**), ZnIn_2_S_4_ (**e**), and 40In_2_O_3_/ZnIn_2_S_4_ (**f**); TEM images of 40In_2_O_3_/ZnIn_2_S_4_ (**f**,**g**); (**h**) Elemental mapping of 40In_2_O_3_/ZnIn_2_S_4_ composite.

The microstructures of MIL, MIL-derived In_2_O_3_, ZnIn_2_S_4_, and 40In_2_O_3_/ZnIn_2_S_4_ composite were characterized using SEM. As shown in Figure 4a, MIL exhibits a rod-like structure with a smooth and uniform surface, and the rod width is approximately 3 μm. Figure 4b–d show the SEM images of the derived In_2_O_3_ products after calcination. The In_2_O_3_ retains the rod-like morphology of MIL but develops a hollow structure with abundant surface voids, possibly due to the removal of organic ligands during combustion [15]. Figure 4e displays the SEM image of ZnIn_2_S_4_, revealing a flower-ball structure composed of nanoplates. This nanoplate structure endows ZnIn_2_S_4_ with strong light absorption performance and provides numerous active sites [21]. Figure 4f shows the SEM image of the 40In_2_O_3_/ZnIn_2_S_4_ composite, where the surface of In_2_O_3_ becomes roughened following the in situ growth of ZnIn_2_S_4_. Combined with Figure 4g, it is evident that a multi-layered ZnIn_2_S_4_ nanoplate was loaded on the surface of In_2_O_3_. Figure 4h presents the EDS spectra of the 40In_2_O_3_/ZnIn_2_S_4_ composite, indicating the uniform distribution of In, O, Zn, and S elements on the surface, confirming the successful composite of ZnIn_2_S_4_ on the hollow tubulous-like In_2_O_3_.

**Figure 5:**
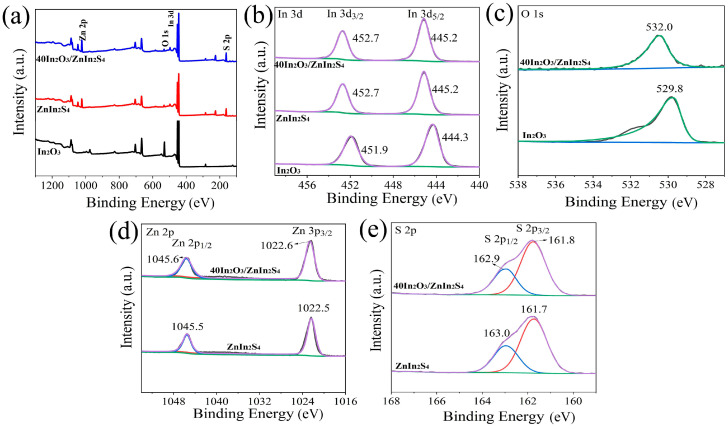
The full XPS survey spectra of In_2_O_3_, ZnIn_2_S_4_, and 40In_2_O_3_/ZnIn_2_S_4_ (**a**); high-resolution XPS spectra of In3d (**b**), O1s (**c**) Zn2p (**d**), and S2p (**e**).

XPS was employed to analyze the surface composition and chemical states of the derived In_2_O_3_, ZnIn_2_S_4_, and 40In_2_O_3_/ZnIn_2_S_4_. Figure 5a presents the XPS survey spectra of the three samples. It can be observed that both the prepared ZnIn_2_S_4_ and 40In_2_O_3_/ZnIn_2_S_4_ exhibit characteristic peaks corresponding to Zn2p, In3d, and S2p. In the In3d orbital XPS spectra (Figure 5b), all three samples show two distinct peaks. For ZnIn_2_S_4_, the peaks at 452.7 eV and 445.2 eV are attributed to the In3d_3/2_ and In3d_5/2_ orbitals of ZnIn_2_S_4_, respectively. Similarly, for 40In_2_O_3_/ZnIn_2_S_4_, peaks corresponding to In3d_3/2_ and In3d_5/2_ appear at the same binding energies as ZnIn_2_S_4_, indicating the substantial in situ growth of ZnIn_2_S_4_ on the In_2_O_3_ carrier. In contrast, the binding energies of In3d_3/2_ and In3d_5/2_ in pure In_2_O_3_ are located at 451.9 eV and 444.3 eV, respectively, indicating the presence of In^3+^ in the samples. Figure 5c displays the orbital peaks of O1s. In In_2_O_3_, the O1s peak appears at a binding energy of 529.8 eV, corresponding to lattice oxygen. In contrast, the high binding energy peak at 532.0 eV for the In_2_O_3_/ZnIn_2_S_4_ composite is typically attributed to chemisorbed surface oxygen [22,23]. As shown in Figure 5d, Zn2p in ZnIn_2_S_4_ exhibits two characteristic peaks: Zn2p_1/2_ at 1045.5 eV and Zn2p_3/2_ at 1022.5 eV. For the 40In_2_O_3_/ZnIn_2_S_4_, the Zn2p orbital peaks are observed at 1045.6 eV (Zn2p_1/2_) and 1022.6 eV (Zn2p_3/2_), indicating a slight shift towards higher binding energies of 0.1 eV compared to ZnIn_2_S_4_. From the high-resolution spectra of S2p (Figure 5e), it can be observed that for ZnIn_2_S_4_, the S2p_1/2_ and S2p_3/2_ orbital peaks are located at 163.0 eV and 161.7 eV, respectively. For the 40In_2_O_3_/ZnIn_2_S_4_, the characteristic peaks of S2p_1/2_ and S2p_3/2_ orbitals are found at 162.9 eV and 161.8 eV, respectively. Sulfur exists in the S^2-^ state in both ZnIn_2_S_4_ and 40In_2_O_3_/ZnIn_2_S_4_ [22]. For the 40In_2_O_3_/ZnIn_2_S_4_, there is a negative shift in the binding energy of S compared to that of ZnIn_2_S_4_. The shift in binding energies of the Zn and S orbital peaks indicates strong interactions between In_2_O_3_ and ZnIn_2_S_4_ [24,25]. Simultaneously, it suggests that due to the interfacial coupling between In_2_O_3_ and ZnIn_2_S_4_, there is charge transfer occurring between the two phases to achieve a new equilibrium. Interfacial tight binding can promote the separation and migration of photogenerated charge carriers, reduce the electron–hole recombination rate, and enhance the charge transfer rate in photocatalytic reactions, thereby improving the activity of In_2_O_3_/ZnIn_2_S_4_ composite photocatalysts [26]. XPS analysis confirms the chemical states of In, Zn, S, and O elements in the samples as In^3+^, Zn^2+^, S^2−^, and O^2−^, respectively, verifying the presence of In_2_O_3_ and ZnIn_2_S_4_ in the composite.

### 3.2. Analysis of Photocatalytic Degradation of MO and Sterilization of P. aeruginosa

**Figure 6:**
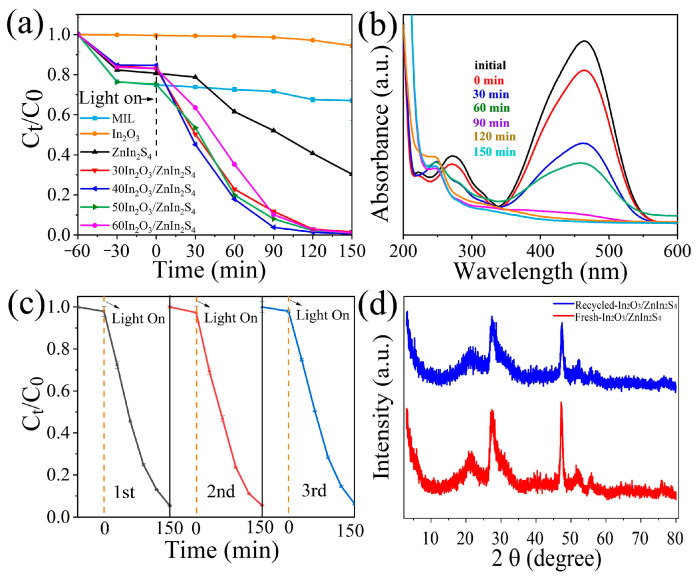
(**a**) Photodegradation of MO by MIL, In_2_O_3_, ZnIn_2_S_4_, and In_2_O_3_/ZnIn_2_S_4_ composites; (**b**) the changes in UV absorbance of methyl orange during the degradation process by 40In_2_O_3_/ZnIn_2_S_4_; (**c**) cycling degradation of 40In_2_O_3_/ZnIn_2_S_4_; (**d**) XRD patterns of 40In_2_O_3_/ZnIn_2_S_4_ after three cycles under visible light irradiation; (**d**) the changes in the XRD pattern of 40In_2_O_3_/ZnIn_2_S_4_ after degrading methyl orange.

The photocatalytic activity of the composite was evaluated by assessing the degradation performance of MO solution under visible light irradiation. As shown in Figure 6a, the photocatalytic material reached adsorption–desorption equilibrium after 1 h. Under simulated visible light irradiation for 150 min, MIL, In_2_O_3_, and ZnIn_2_S_4_ exhibited degradation rates of 33.0%, 5.6%, and 69.7% respectively. The 40In_2_O_3_/ZnIn_2_S_4_ composite showed the highest degradation efficiency, reaching 99.5%, which was 3.0, 17.9, and 1.4 times higher than that of the pure MIL, In_2_O_3_, and ZnIn_2_S_4_, respectively. Other ratios of composites also significantly enhanced the degradation of MO, indicating that the combination of In_2_O_3_ and ZnIn_2_S_4_ facilitates the separation of photogenerated charge carriers, thereby improving its degradation performance. As depicted in Figure 6b, under visible light exposure, the absorbance of MO at different illumination times decreased gradually for the 40In_2_O_3_/ZnIn_2_S_4_, particularly at the peak intensity around 465 nm, indicating the effective degradation of MO.

To assess the stability of the 40In_2_O_3_/ZnIn_2_S_4_ composite in degrading MO solution through recycling tests of recovered samples, as shown in Figure 6c, its degradation efficiency remained consistently high after three cycles of testing. This indicates excellent stability during the photocatalytic process and demonstrates good repeatability. Additionally, the XRD diffraction peaks of the 40In_2_O_3_/ZnIn_2_S_4_ composite showed no significant changes before and after degradation, suggesting the robust structural stability of the composite photocatalyst before and after the reaction.

**Figure 7:**
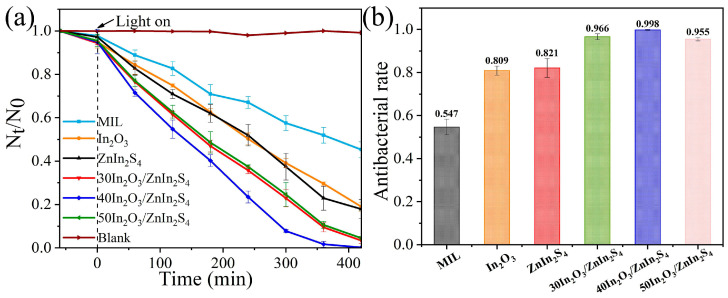
(**a**) Survival rates and (**b**) antimicrobial rates of MIL, In_2_O_3_, ZnIn_2_S_4_, and In_2_O_3_/ZnIn_2_S_4_ composites against *P. aeruginosa*.

To evaluate the antibacterial and antifouling performance of In_2_O_3_, ZnIn_2_S_4_, and In_2_O_3_/ZnIn_2_S_4_ composites, their antibacterial efficiency against the typical marine fouling microorganism *P. aeruginosa* (5.9 × 10^6^ cfu·mL^−1^) under visible light was assessed. In Figure 7a, it is evident that there was minimal change in the bacterial count in the control group, indicating that light exposure had a minimal effect on the activity of *P. aeruginosa*. Compared to the individual photocatalysts MIL, In_2_O_3_, and ZnIn_2_S_4_, the In_2_O_3_/ZnIn_2_S_4_ composite exhibited enhanced antibacterial performance, with 40In_2_O_3_/ZnIn_2_S_4_ performing the best. As shown in Figure 7b, after visible light irradiation of 80 mW cm^−2^ for 420 min, 40In_2_O_3_/ZnIn_2_S_4_ achieved an antibacterial rate of 99.8% against *P. aeruginosa*, which is significantly higher compared to that of MIL, In_2_O_3_, and ZnIn_2_S_4_ by 45.1%, 18.9%, and 17.7%, respectively. The antibacterial rates of the 30In_2_O_3_/ZnIn_2_S_4_ and 50In_2_O_3_/ZnIn_2_S_4_ composites were 96.6% and 95.5%, respectively, also notably higher than those of the individual materials. The excellent antibacterial efficacy of the In_2_O_3_/ZnIn_2_S_4_ composite demonstrates its potential for highly effective antibacterial action in real marine environments.

### 3.3. Mechanism Analysis of the Promotion of Photocatalytic Performance

**Figure 8:**
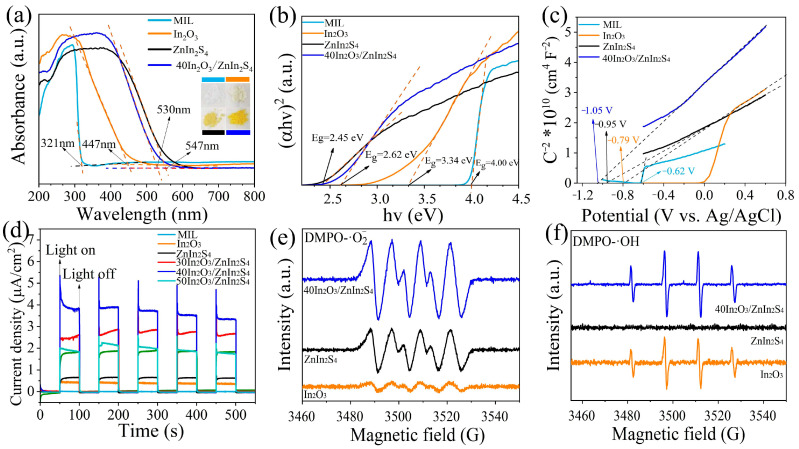
(**a**) UV–Vis diffuse reflection absorbance spectra, (**b**) Tauc plots, (**c**) Mott–Schottky curves of MIL, In_2_O_3_, ZnIn_2_S_4_, and 40In_2_O_3_/ZnIn_2_S_4_, (**d**) photocurrent response curves of MIL, In_2_O_3,_ ZnIn_2_S_4_, and In_2_O_3_/ZnIn_2_S_4_ composites, (**e**,**f**) EPR spectra of In_2_O_3_, ZnIn_2_S_4_, and 40In_2_O_3_/ZnIn_2_S_4_.

Figure 8a shows the UV–Vis diffuse reflectance spectra of MIL, In_2_O_3_, ZnIn_2_S_4_, and In_2_O_3_/ZnIn_2_S_4_ composite. It can be observed that pure MIL has an absorption threshold at 321 nm, while the derived In_2_O_3_ shows an increased absorption threshold at 447 nm, indicating enhanced light absorption performance due to high-temperature derivation from MIL. ZnIn_2_S_4_ exhibits a larger absorption threshold at 547 nm, which further increases the threshold of In_2_O_3_ to 530 nm, leading to enhanced light absorption intensity. Figure 8b presents the band gaps (*E*_g_) of MIL, In_2_O_3_, ZnIn_2_S_4_, and 40In_2_O_3_/ZnIn_2_S_4_ calculated from the Tauc plot [27]. It can be seen that the band gaps of MIL, In_2_O_3_, ZnIn_2_S_4_, and 40In_2_O_3_/ZnIn_2_S_4_ are 4.00 eV, 3.34 eV, 2.45 eV, and 2.62 eV, respectively. Compared to MIL, In_2_O_3_ has a lower band gap, indicating increased sensitivity to light. After the composite with ZnIn_2_S_4_, the band gap of In_2_O_3_ further decreases, enhancing its photosensitivity, consistent with the diffuse reflectance spectroscopy (DRS) results. The Mott–Schottky test results indicate that MIL, In_2_O_3_, and ZnIn_2_S_4_ samples all exhibit positive slopes, indicating that these materials are n-type semiconductors. Therefore, the intersection of the tangent to the curve with the X-axis gives the flat band potential (*E*_fb_) of the materials [26,27]. Figure 8c shows the flat band potentials (*E*_fb_) for MIL, In_2_O_3_, ZnIn_2_S_4_, and 40In_2_O_3_/ZnIn_2_S_4_, which are −0.62 V, −0.79 V, −0.95 V, and −1.05 V (vs. Ag/AgCl), respectively. For n-type semiconductors, *E*_fb_ is approximately equal to *E*_CB_. Thus, the *E*_CB_ values for MIL, In_2_O_3_, ZnIn_2_S_4_, and 40In_2_O_3_/ZnIn_2_S_4_ are −0.62 V, −0.79 V, −0.95 V, and −1.05 V (vs. Ag/AgCl), which correspond to −0.42 V, −0.59 V, −0.75 V, and −0.85 V (vs. NHE), respectively. According to the *E*_VB_ = *E*_g_ + *E*_CB_ equation, the valence band edges (*E*_VB_) for MIL, In_2_O_3_, ZnIn_2_S_4_, and 40In_2_O_3_/ZnIn_2_S_4_ are 3.58 V, 2.75 V, 1.70 V, and 1.77 V (vs. NHE), respectively.

Figure 8d shows the photocurrent densities of different samples in 3.5 wt% NaCl solution under simulated sunlight irradiation of 100 mW·cm^−2^. It can be observed that the photocurrent density of the electrode made from pure MIL powder is relatively low, whereas the derived In_2_O_3_ shows a moderate increase in photocurrent density. The In_2_O_3_/ZnIn_2_S_4_ composite exhibits significantly enhanced photocurrent density compared to both In_2_O_3_ and ZnIn_2_S_4_ alone, with 40In_2_O_3_/ZnIn_2_S_4_ showing the highest photoresponse. The increased photocurrent density of the composites indicates that the In_2_O_3_/ZnIn_2_S_4_ heterojunction promotes the efficient separation of photogenerated charge carriers.

To investigate the electron transfer mechanism between In_2_O_3_ and ZnIn_2_S_4_, ESR was used to further explore the generation of radicals in In_2_O_3_, ZnIn_2_S_4_, and 40In_2_O_3_/ZnIn_2_S_4_ [28]. The results, as shown in Figure 8e,f, reveal that after 20 min of visible light irradiation, 40In_2_O_3_/ZnIn_2_S_4_ exhibits higher peaks in ·O_2_^−^ and ·OH signals compared to In_2_O_3_ and ZnIn_2_S_4_ individually, indicating that the heterojunction formed by In_2_O_3_ and ZnIn_2_S_4_ promotes the separation of photogenerated electrons and holes. ESR testing confirms that ZnIn_2_S_4_ does not generate ·OH radicals under visible light irradiation, which correlates with its valence band being lower than the potential of H_2_O/ OH. In contrast, In_2_O_3_ can generate both ·O_2_^−^ and ·OH radicals under light exposure. 

**Figure 9:**
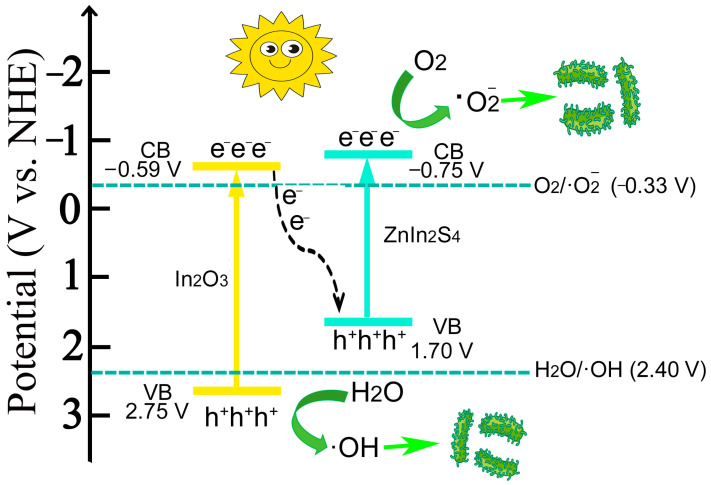
Possible charge transfer mechanism of In_2_O_3_/ZnIn_2_S_4_.

Based on the types and intensities of the generated radicals, the ·O_2_^−^ and ·OH signals of the 40In_2_O_3_/ZnIn_2_S_4_ composite are stronger compared to those of In_2_O_3_ and ZnIn_2_S_4_ alone. This indicates that the heterojunction formed between In_2_O_3_ and ZnIn_2_S_4_ enhances the separation of photogenerated charge carriers. In ESR results, In_2_O_3_ can simultaneously produce ·O_2_^−^ and ·OH under light exposure because its valence band (*E*_VB_ = 2.75 V vs. NHE) is higher than the potential of H_2_O/·OH (*E*_VB_ = 2.40 V vs. NHE), and its conduction band (*E*_CB_ = −0.59 V vs. NHE) is lower than the potential of O_2_/·O_2_^−^ (*E*_CB_ = −0.33 V vs. NHE). Conversely, the valence band of ZnIn_2_S_4_ (*E*_VB_ = 1.70 V vs. NHE) is lower than the potential of H_2_O/·OH; thus, it cannot generate ·OH radicals but can produce ·O_2_^−^ due to its lower conduction band (*E*_CB_ = −0.75 V), capable of reducing O_2_ to generate radicals. Therefore, based on these deductions, it is more likely that a Z-scheme heterojunction forms between In_2_O_3_ and ZnIn_2_S_4_. This Z-scheme heterojunction preserves the highly reductive photogenerated electrons of ZnIn_2_S_4_ and highly oxidative photogenerated holes of In_2_O_3_. Then, this retains the high reducibility of ZnIn_2_S_4_ while leveraging the strong oxidizing capability of In_2_O_3_, enhancing the degradation and sterilization performance. Below is a schematic diagram illustrating the potential charge transfer pathways in the In_2_O_3_/ZnIn_2_S_4_ composite, as shown in Figure 9:

According to the above conclusion (Figure 9), a possible mechanism [29] of the bactericidal action of In_2_O_3_/ZnIn_2_S_4_ is as follows:

In_2_O_3_ → In_2_O_3_ (e^−^ + h^+^)     ZnIn_2_S_4_ → ZnIn_2_S_4_ (e^−^ + h^+^) (1)

In_2_O_3_ (e^−^ + h^+^) + ZnIn_2_S_4_ (e^−^ + h^+^) → In_2_O_3_ (h^+^) + ZnIn_2_S_4_ (e^−^) (2)

ZnIn_2_S_4_ (e^−^) + O_2_ → ·O_2_^−^ (3)

In_2_O_3_ (h^+^) + H_2_O → ·OH (4)

Live bacteria +·O_2_^−^ + ·OH → Dead bacteria (5)

## 4. Conclusions

The hollow tubulous-shaped In_2_O_3_ derived from metal-organic framework (MOF) structure MIL was obtained via calcination, which exhibits an increased specific surface area, reactive sites, light absorption performance, and photoelectric response performance. Adopting this derived In_2_O_3_ as a template, nanoflower-shaped ZnIn_2_S_4_ was in situ-composited on it and obtained the In_2_O_3_/ZnIn_2_S_4_ composite, resulting in enhancing performance in MO degradation and *P. aeruginosa* sterilization. The optimal 40In_2_O_3_/ZnIn_2_S_4_ composite exhibited degradation rates of MO under visible light (80 mW·cm^−2^, 150 min) that were 17.9 and 1.4 times higher than those of the pure In_2_O_3_ and ZnIn_2_S_4_, respectively. And the 40In_2_O_3_/ZnIn_2_S_4_ shows the highest antibacterial performance against *P. aeruginosa*, with an antibacterial rate of 99.8% after visible light irradiation of 80 mW cm^−2^ for 420 min. Even after three cycles of reuse, the 40In_2_O_3_/ZnIn_2_S_4_ maintained high degradation activity without significant structural changes.

SEM, TEM, and XPS characterizations confirmed the tight contact between In_2_O_3_ and ZnIn_2_S_4_. The enhanced photocurrent density further indicates the presence of heterojunction. The ESR analysis of free radicals indicated that the 40In_2_O_3_/ZnIn_2_S_4_ generated both ·O_2_^−^ and ·OH radicals under visible light, whereas ·OH radicals were not detected in ZnIn_2_S_4_ alone, suggesting the presence of a Z-scheme heterojunction between In_2_O_3_ and ZnIn_2_S_4_. The Z-scheme heterojunction preserves the highly reductive photogenerated electrons of ZnIn_2_S_4_ and highly oxidative photogenerated holes of In_2_O_3_, simultaneously enhancing the separation of photogenerated charge carriers.

This in situ growth of ZnIn_2_S_4_ on MIL-derived In_2_O_3_ produces an efficient composite for photocatalytic degradation and sterilization, offering guidance for applications in photocatalytic antimicrobial and antifouling treatments. Additionally, the synthesis conditions of the materials in this article are relatively mild, the raw materials are widely sourced, and only a small amount is needed to achieve significant degradation and bactericidal effects. At the same time, the physicochemical properties are stable, allowing for multiple cycles of use, which better meets the cost control requirements in practical applications and demonstrates considerable potential for application.

## Data Availability

The data are contained within the article.
